# The relevance of the history of biotechnology for healthcare: Teaching students how biotechnology and medicine have been closely entwined during the past century highlights how both fields have inspired and driven each other

**DOI:** 10.1038/s44319-024-00355-8

**Published:** 2025-01-02

**Authors:** Maurizio Bifulco, Erika Di Zazzo, Alessandra Affinito, Cristina Pagano

**Affiliations:** 1 Department of Molecular Medicine and Medical Biotechnology, University of Naples “Federico II”, Naples, Italy; 2 Department of Medicine and Health Sciences “V. Tiberio”, University of Molise, 86100 Campobasso, Italy; 3 UOC Laboratorio Analisi, Ospedale “A. Cardarelli”, 86100 Campobasso, Italy

**Keywords:** Biotechnology & Synthetic Biology, History & Philosophy of Science, Molecular Biology of Disease

## Abstract

Given how biotechnology has revolutionized medical research, drug development and treatment across fields like precision medicine, cancer therapy, and vaccine development, courses on the History of Biotechnology in academic curricula can inspire future scientists and clinicians to continue improving human health and well-being.

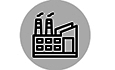

Biotechnology is a multidisciplinary science that uses biological systems and processes to address scientific, technological, and healthcare challenges (Martin et al, 2021; Thorsteinsdottir et al, 2011). Its roots date back to ancient times, when humans selected plants for cultivation, domesticated animals and used microorganisms to produce bread, wine, and beer. At that time, there was no scientific knowledge available—nor the means to acquire this knowledge—about the underlying processes and molecules, and people relied on experience for breeding of plant and animal species and fermentation. Throughout history, scientists have tried to explain these phenomena. However, it was only during the second half of the 19th century, when biotechnology gained a rigorous scientific basis: Louis Pasteur identified and isolated yeast as the agent responsible for the transformation of must into wine, Gregor Mendel formulated the laws of genetics and Friedrich Miescher discovered nucleic acids.

During the past four decades, biotechnology has evolved into a sophisticated discipline, applying the power of molecular biology and genetic engineering to produce new products and novel processes to continue revolutionising our live (Abuduxike and Aljunid, 2012; Lokko et al, 2018). Scientists began to understand and use biological systems to develop new diagnostic tools and therapeutic strategies. This approach has paved the way for innovative treatments and technologies previously considered unachievable. Biotechnology, indeed, is currently employed in all medical fields to satisfy the constant need to improve the health and quality of life for humans.

“During the past four decades, biotechnology has evolved into a sophisticated discipline […] to produce new products and novel processes to continue revolutionising our lives.”

While the history of medicine became a formal field in 1892, when Valeriu Lucian Bologa (1892–1971), a Romanian professor, gave the first lecture of Medicine History (Barsu, 2017), Biotechnology History still suffers from a lower awareness and lack of appreciation. Nonetheless, if Medicine History provides important insights about disease etiology and treatment, Biotechnology History gives essential information on the tools to diagnose, treat, and monitor diseases. In addition, the discoveries and the experiences of the past century highlight that the history of medicine and biotechnology are intertwined and interconnected. These two fields show dual and changing roles, with biotechnology being the driving force behind medicine’s progress and at the same time medicine driving research and development in biotechnology advancements.

“… if Medicine History provides important insights about disease etiology and treatment, Biotechnology History gives essential information on the tools to diagnose, treat and monitor diseases.”

## A course on the history of biotechnology

Understanding the history and achievements of biotechnology, along with its continuous impact on discovery, diagnosis and treatment, is essential for medical scientists to understand the importance of both fields of medicine and biotechnology to improve healthcare. Moreover, students may learn from historical courses and examples throughout the history of medicine and biotechnology to encourage their creativity in future disease management. In the academic year 2023/2024, one of the authors and the general Pathology Dean of “Federico II” University in Naples, Italy, Maurizio Bifulco, proposed the introduction of the History of Medicine and Biotechnology course to the medical faculty. This proposal was unanimously accepted and it represents the first course experimentally introduced in Europe; we hope that it can be also be adapted at other universities.

The main goal of the course, given by researchers from the university, is to teach students the key discoveries that marked the progression of medical art and biotechnology. To this end, the History of Medicine and Biotechnology covers several topics including: the pioneering medieval medical schools, recombinant DNA, the development of recombinant insulin and its impact on diabetes management, the groundbreaking advances in AIDS therapy and diagnosis, the history of cell cultures and stem-cell research, the development of monoclonal antibodies, the Human Genome Project, Assisted Reproduction and gene therapy. The lecturers adopted a problem-oriented, situational teaching style to stimulate the interest of the students and to encourage them to acquire new knowledge and use it in the context of their future professional activities. In addition, teachers implemented different methodological approaches such as the use of primary sources—examples of historical texts, medical journals, and medical prescriptions—to depict the historical thoughts and practices at different time periods, and digital tools including music videos and short films to achieve a more immersive learning experience.

Additional thematic lectures by invited speakers helped to deepen knowledge in specialized areas and bear witness to the scientific progress. For instance, an invited speaker gave a lecture about the mapping and sequencing of the human genome. More lectures and visits have already been arranged for the next year along with visits to medical museums. In this regard, the course will take advantage from the re-opening of the historical pharmacy of the Hospital “Incurabili” in the center of Naples after 5 years of restoration. “Incurabili” means uncurable patients, and here the most serious patients of the city were hospitalized starting from 1519. Students will appreciate the history of medicine through seeing old surgical instruments, ancient remedies, and the storytelling of successes and failures, aimed to improve medical and scientific progress, everything surrounded by paintings and arts.

The interdisciplinary course should help students to acquire five main skills. First, to understand the nature of scientific investigation which is a continuous relationship between theoretical construction and experimental activity; second, to adopt a critical approach and awareness of the scientific and technological issues of current society; third, to understand that science does not construct definitive dogmas but rather interpretations that are amenable to modification and falsification; and lastly, to be aware of the potential limits of technologies in the cultural and social context in which they are applied. The final test was an oral examination to assess the students’ ability to apply such knowledge in contexts that simulate or describe real situations. In addition, it also evaluated the ability to make intra and interdisciplinary connections.

The History of Medicine and Biotechnology course received positive feedback from the students who were excited to learn the relevance of drawing on history to obtain novel ideas for the present and the future. Thus, given the positive response, we are convinced that the introduction of this course in the curriculum of future biotechnologists and scientists is essential to prepare both of them for their careers. In addition, it is relevant to know how all the innovative technologies and successes developed throughout history have raised a series of social and ethical issues.

## A brief timeline of modern biotechnology

Modern biotechnology emerged in the early 20th century. A turning point came in 1928 with the discovery of penicillin by Alexander Fleming who accidentally observed that a mold, later identified as a rare strain of *Penicillium Notatum*, inhibited the growth of *Staphylococcus* colonies on a Petri dish. Fleming obtained an extract from the mold, naming its active agent penicillin, and determined that it has an antibacterial effect on staphylococci and other gram-positive bacteria. However, several attempts made by Fleming’s group to purify penicillin failed; he eventually published his findings in the *British Journal of Experimental Pathology* in June 1929, and referred in an elusive manner to penicillin’s potential therapeutic benefits (https://www.acs.org/education/whatischemistry/landmarks/flemingpenicillin.html). The onset of World War II forced scientists and engineers to collaborate in developing large-scale culture of *Penicillium* to quickly produce penicillin, facing the need to cure wounded soldiers and civilians (Renneberg and Loroch, 2016). This effort has led to the improvement of fermentation technology and made penicillin widely available.

“The onset of World War II forced scientists and engineers to collaborate in developing large-scale culture of *Penicillium* to quickly produce penicillin…”

The discovery of the structure of DNA by James Watson and Francis Crick and the development of DNA synthesis by Arthur Kornberg in the 1950s marked a watershed moment and led to the birth of genetic engineering and recombinant DNA (rDNA) technology (Cohen et al, 1973). These enabled David Goeddel’s group at Genentech, the first biotech company (Liao and Wang, 2023), in 1978, to produce recombinant human insulin in *Escherichia coli*. It was the first in vitro animal-free, non-immunogenic recombinant protein and quickly became the standard therapy for treating diabetes patients worldwide (Falcetta et al, 2022). Genentech’s success inspired many new paradigms for disease diagnosis and treatment as well as the start of many other biotechnology companies.

The 1990s witnessed the Human Genome Project and the first gene therapies in humans, notably Alain Fischer’s successful cure of a rare and severe immune deficiency (Hacein-Bey-Abina et al, 2002). In 1995, the first genome of a living organism, *Haemophilus influenzae*, was sequenced and 2 years later, the cloning of the sheep Dolly demonstrated the potential of cellular manipulation in mammals. The growing knowledge about recombinant DNA technology further paved the way for genome editing, which achieved its first tangible results in 2005 by using zinc finger nucleases (ZFNs), and the transcription activator-like effector nucleases (TALENs) in 2010. Finally, CRISPR-Cas gene-editing technology, based on a natural bacterial defense mechanism, was developed in 2013. It allows the precise manipulation of DNA by adding, deleting or replacing specific genes or individual nucleotides in living cells (Doudna and Charpentier, 2014).

During the same period, mRNA attracted interest as a therapeutic tool. In late 1987, Robert Malone took the first steps toward RNA therapeutics by mixing mRNA with lipidic droplets that were able to enter living human cells. However, until the late 2000s, the development of mRNA therapies was held back by RNA’s instability and high production costs. Nonetheless, the idea of mRNA vaccines gained traction in oncology, albeit as a therapeutic agent rather than to prevent disease. Several scientists and start-up companies explored the technology to combat cancer through the expression of mRNA-encoded proteins to stimulate the immune system against tumor cells. In 2008, both Novartis and Shire established mRNA research units, the former focused on vaccines, the latter on therapeutics.

Moderna was one of the companies that built on this work and, by 2015, it had raised more than US$1 billion on the promise of harnessing mRNA to restore missing or defective disease-causing proteins. When that plan faltered, Moderna chose to prioritize vaccines. When COVID-19 struck, Moderna quickly created a prototype vaccine within days after the virus’s genome sequence became available and started human trials within less than ten weeks. BioNTech partnered with Pfizer in March 2020, and clinical trials moved at a record pace, going from first-in-human testing to emergency approval in less than 8 months (Dolgin, 2021). Both vaccines use modified mRNA formulated in lipid nanoparticles (LNPs), containing sequences that encode a form of the SARS-CoV-2 spike protein to induce protective immunity. The mRNA vaccines greatly contributed to fight the pandemic spread of SARS-CoV-2 virus infection.

Precision medicine is the main application of biotechnology in healthcare to tailor disease prevention, perform diagnosis and treatment by considering the specific genetic and molecular characteristics of each patient. The idea traces back to Sir William Osler who said: “It is much more important to know what sort of a patient has disease than what sort of a disease a patient has,” and to Werner Kalow’s work on Pharmacogenetics in 1962 (Jones, 2013). It gained significant attention in 2015, when US President Barack Obama launched a precision-medicine initiative with the goal to deliver targeted treatments according to the unique biology of each patient, and thereby optimize drug efficacy. Two meaningful successes in this field include the approval of Trastuzumab (Herceptin; Genentech) in 1988 for treating HER2-positive metastatic breast cancer patients, and Imatinib (Gleevec; Novartis) in the early 2000s to treat chronic myeloid leukemia (CML) by targeting BCR-ABL tyrosine kinase (Jorgensen, 2019).

History clearly demonstrates that biotechnology has had a crucial impact on cancer treatment. Indeed, biotechnological advances give cancer patients new hope by developing less harmful and more effective treatment options compared to conventional therapies. Innovative cancer therapeutics include: immune checkpoint inhibitors to increase the immune system’s ability to recognize and attack cancer cells; CAR T-cell therapy that uses genetically modified T-cells from the patient’s blood to better recognize and attack cancer cells; targeted therapy that specifically focus on specific mutations or proteins involved in cancer growth and metastasis; gene therapy to correct or replace defective genes involved in cancer; and nanoparticle-based therapy, an innovative approach to deliver drugs directly to cancer cells (Liao and Wang, 2023).

“… biotechnological advances give cancer patients new hope by developing less harmful and more effective treatment options compared to conventional therapies.”

**Figure 1:**
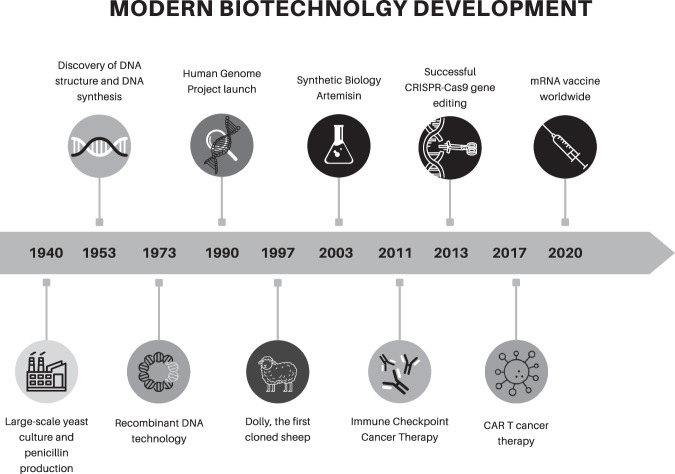
Timeline of major development milestones in modern biotechnology. The image represents the various discoveries made in the biotechnology field since 1940.

In summary, modern biology and biotechnology have progressed with crucial discoveries and innovations in many different fields (Fig. 1). Advances in antibiotics, genetics, RNA therapeutics and precision medicine, along with AI and Big Data analysis, promise more effective and personalized future medical interventions.

## Critical challenges

Over the years, concerns about biotechnology have been inflamed by suspicions that science is merely a tool for a technological imperative, that because something can be done, it should be done. As modern biology and its applications expanded, so did the demands to control how this knowledge will be used. Starting with the Human Genome Project, ethicists, scientists, and lawyers began to work together to assess not only what we can do, but also what we should do. Indeed, the application of specific innovations, such as cloning, whole-genome sequencing or gene editing raises significant ethical, legal, and societal issues about the safety and potential impact of genetically modified organisms and possible misuse. Concerns also exist about the long-term environmental consequences of modifying organisms’ genomes.

Another important challenge is the clinical translation of biotechnology and the difficulties in processing these into commercial products. Indeed, only a few biotech advances have so far resulted in new healthcare tools and treatments. The bench-to-bedside translation involves several stages beyond discovery and clinical development: the search for funding, the difficulties in clinical trial design and execution, regulatory approvals, market acceptance, and competition with other healthcare industries. These economic and regulatory factors in addition to the above-mentioned ethical, legal, and societal issues play important roles in regard to how rapidly and efficiently biotechnology can improve healthcare—the pace with which industry developed and advanced the mRNA vaccines against SARS-CoV2 is an important lesson.

“… economic and regulatory factors in addition to the above-mentioned ethical, legal and societal issues play important roles in regard to how rapidly and efficiently biotechnology can improve healthcare…”

## Conclusion

History shows the potential of biotechnology to drive innovation in both medicine and pharmacology and that it has made and continues to make transformative advances in healthcare, by transforming the discovery, development, and manufacturing of drugs and other therapies. Medicine embraces these advances and applies them in clinical practice, revolutionizing fields such as biopharmaceutics and biologics, targeted drugs, personalized medicine, and gene therapy.

Biotech companies, in particular small and medium enterprises (SMEs), have become major players in this process: great examples are Germany-based BioNTech and US-based Moderna and their contributions to fighting the COVID-19 pandemic. The great importance of SMEs in medical development and economic growth has been widely recognized. To support their taking risks on cutting-edge technologies, governments offer specific funding sources such as the Horizon Europe program for European SMEs and the Small Business Innovation Research Program (SBIR) in the USA.

In summary, biotechnology is now a cornerstone of medical and pharmacological evolution, giving physicians and patients new treatment options. Although the field is often harnessed to ethical issues that at times hamper advancements, it is evident that biotechnology has brought humanity well-being and health, and its impact will only increase in the future. Highlighting the great efforts that have been made and the goals that have been reached so far, demonstrates students of medicine and biology the importance of technology in medical progress. Hence the argument for adopting a course on the History of Biotechnology for university curricula to complement courses on the history of medicine: a deeper knowledge of how both fields have inspired each other and drove progress in healthcare may train future scientists and clinicians to further push improve human well-being.

## Supplementary information

Peer Review File
