# Supplementary material for: The relevance of the history of biotechnology for healthcare: Teaching students how biotechnology and medicine have been closely entwined during the past century highlights how both fields have inspired and driven each other
Source: EMBO Rep. 2025 Jan 2;26(2):303–6. doi: 10.1038/s44319-024-00355-8 (PMC11772680; doi:10.1038/s44319-024-00355-8)
Supplement: Supplementary file 1 — Peer Review File [file 44319_2024_355_MOESM1_ESM.pdf]

EMBO reports

The relevance of the history of biotechnology for healthcare

Maurizio Bifulco, Erika Di Zazzo, Alessandra Affinito, and Cristina Pagano

Corresponding author(s): Cristina Pagano (cristina.pagano@unina.it) , Maurizio Bifulco (maubiful@unina.it)

Review Timeline:

Submission Date:	1st Dec 24
Accepted:	13th Dec 24

Editor: Holger Breithaupt

Transaction Report: This article was editorially reviewed.
